# Alterations in gut microbiota characteristics along a type 2 diabetes risk gradient linked with family history

**DOI:** 10.1080/29933935.2025.2527766

**Published:** 2025-07-22

**Authors:** Oscar Gitton-Quent, Mathilde Sola, Nicolas Maziers, Anne Hiol, Nicolas Dechamp, Emmanuelle Le Chatelier, Mathilde Touvier, Pilar Galan, Aymeric David, Christian Morabito, Alexandre Famechon, Benoit Quinquis, Mahendra Mariadassou, Patrick Veiga, Joel Dore, Magali Berland, Melanie Deschasaux-Tanguy

**Affiliations:** a Université Paris-Saclay, INRAE, MGP, Jouy-en-Josas, France; b Hospital Center Sud Francilien, Intensive Care Unit, JCorbeil-Essonnes, France; c Université Sorbonne Paris Nord and Université Paris Cité, INSERM, INRAE, CNAM, Centre for Research in Epidemiology and StatisticS (CRESS), Nutritional Epidemiology Research Team (EREN), Bobigny, France; d Université Paris-Saclay, INRAE, MaIAGE, Jouy-en-Josas, France; e Université Paris-Saclay, INRAE, Micalis, Jouy-en-Josas, France

**Keywords:** Type 2 diabetes, human gut microbiota, nutrition, microbial guilds, shotgun metagenomics

## Abstract

Type 2 diabetes (T2D) is a major global health issue, with growing evidence linking it to gut microbiome changes. However, whether these alterations precede T2D onset and act as predictors, risk factors, or contributors remains unclear. This study analyzed the gut microbiota of 192 individuals from the French NutriNet-Santé cohort, divided into four groups: non-T2D adults with no (*n* = 47), one (*n* = 48), or two (*n* = 51) T2D-affected parents, and T2D-affected adults (*n* = 46). A progressive microbiota shift was observed in non-T2D groups based on parental history, converging toward the T2D profile. Changes included altered enterotype distribution, increased oral-associated species, disrupted ecological networks, and a shift in Gram-positive-to-negative ratios. Notably, *Prevotella copri* abundance increased, alongside bacteria potentially enhancing branched-chain amino acid (BCAA), lipopolysaccharide (LPS), and acetate production. Diet also influenced microbiota patterns, with sweet product intake, vitamin levels, and copper/zinc ratios playing roles. A gradual microbiome transition from non-diabetic to T2D participants underscores its association with family history-based risk. While these shifts may reflect or drive T2D progression, further studies are needed to confirm these findings and explore their potential for preventive strategies.

## Introduction

Type 2 diabetes (T2D) is a rapidly expanding metabolic disease, currently affecting approximately 6% of the global population^1^ and characterized by hyperglycemia, primarily due to insulin resistance. In this condition, muscle, fat, and liver cells become less responsive to insulin, resulting in elevated blood glucose levels. This leads to overactivation of insulin-producing beta cells and a diminished capacity for glucose absorption by muscle cells. Consequently, a vicious cycle of glucose accumulation occurs, ultimately causing beta cell exhaustion and muscle cell insensitivity to insulin. Both host factors, including the genetic predispositions and gut microbiota composition, and environmental factors such as dietary habits, significantly influence its onset and progression.^2^

Studies of the concordance of T2D prevalence in homozygous and heterozygous twins have made it possible to calculate a heritability rate for the disease of 61–78%, taking into account age, BMI and geographical origin.^3^ Similarly, having one or two parents affected by the disease can significantly increase the risk of developing T2D, with a risk multiplied by 2.5 and 4, respectively.^4^ Despite the influence of heredity on the risk of developing T2D, the link with genetics is difficult to establish with more than 500 independent loci found to be associated with the disease. Indeed, only a fraction of these loci is found in several studies and can be explained from a mechanistic point of view via their impact on the functioning of the liver or islets of Langerhans.^5^ Thus, in addition to genetics, exogenous factors such as lifestyle, diet and environmental pollution represent a significant part of the possible causes of T2D development, which can take years to be establish.^6^ During this period, a person can first go through an asymptomatic phase known as pre-diabetes or impaired glucose tolerance, a consequence of which being to increase the risk of developing T2D by up to 70%.^7^ This state can be identified by blood measurements of various criteria like glycated hemoglobin (HbA1C) or glycemia for which elevated levels, intermediate between non-T2D and diabetic individuals, are observed.^8^

A growing literature supported by large cohorts^9^ and multi-cohort studies^10^ is suggesting that T2D and pre-diabetes status are related to specific characteristics in the composition of the gut microbiota when compared to non-T2D individuals. The gut microbiota is an ecosystem of microorganisms residing in the gastrointestinal tract, playing a critical role in health by stimulating the immune system, protecting against pathogenic bacteria, aiding in nutrient digestion, and producing essential metabolites such as vitamins, amino acids, and short-chain fatty acids. However, dysbiosis or alterations in microbiota composition can lead to issues like chronic inflammation and the presence of pathogenic elements. In pre-diabetics, the intestinal microbiota has shown an enrichment in *Streptococcus* or *Escherichia* species, whereas *Lactobacillus*, *Collinsella* species or *Ruminococcus gnavus* were enriched in diabetic compared with non-T2D individuals. In turn, symbiotic bacterial species including *Faecalibacterium pausnitzii*, *Akkermancia muciniphila* and *Roseburia sp* were depleted in pre-diabetes and T2D.^11,12^

Beyond these distinct compositions between groups, which do not allow us to conclude on the causal relationship between alterations in the microbiota and T2D, mechanistic studies have highlighted the importance of the intestinal microbiota in disease development.^12^ For example, branched-chain amino acids (BCAAs) synthesis activates mTOR1 pathway and inhibits lipolysis in adipose cells, resulting in an increase in insulin resistance.^13^ The microbiota’s actions can also affect more global mechanisms such as inflammation. For example, the production of lipopolysaccharides (LPS) and their uptake into the circulation can stimulate TLR4-dependent inflammation, resulting in phosphorylation of the insulin receptor substrate and therefore an increase in insulin resistance.^14^ Similarly, the intestinal microbiota is capable of metabolizing primary bile acids into secondary bile acids, which may impact T2D by regulating glucose and lipid metabolism and GLP-1 secretion.^15^

The relationship between T2DM and the gut microbiota has been demonstrated both in terms of composition and functional potential by experiments such as the transfer of fecal microbiota from healthy donors to T2DM patients over several weeks.^16^ However, the contribution of the intestinal microbiota to the development of the disease, when compared to other determinants such as genetics and the environment, has been little studied. Nevertheless, a recent study has confirmed the small but significant contribution of the intestinal microbiota in predicting the risk of T2D.^17^ Modifications of the gut microbiota in T2D could be induced by lifestyle, including diet, and therefore have environmental origins. However, these modifications could also result from some genetic heritability of gut microbiome characteristics, mother-to-child transmission of the intestinal microbiota at birth, but also to the standardization of ecosystems between household members,^18^ further emphasized in a recent review,^19^ suggesting that part of the heritability of T2D could be carried by the intestinal microbiota.

The aim of this research was to explore the impact of an increased risk of T2D linked to family history on gut microbiota profiles, untangling the influence of diet and lifestyle.

## Material and methods

### Study population and data collection

Participants were recruited from the NutriNetSanté cohort,^20^ an ongoing web-based population study launched in 2009 to explore associations between nutrition and health. The study collects detailed participant data through regular online questionnaires accessible via a dedicated platform. Every year, participants complete questionnaires on sociodemographic characteristics and lifestyle factors, physical activity (International Physical Activity Questionnaire, IPAQ^21^), health (including medication use) and anthropometric measurements.^22^ Diet intake was assessed every 6 months through repeated series of three validated 24-hour dietary records (two week days and one weekend day over a two-weeks period).^23–25^ Participants reported all foods and beverages consumed within 24 hours. Portion sizes were assessed through validated photographs, standard serving sizes or directly in grams or milliliters. Nutrient and energy intakes, including alcohol, were calculated from the NutriNet-Santé food composition table, comprising >3500 generic food items.

### Stool sample collection

In 2021, participants aged between 45 and 65 years old were invited to enroll in the *Homo symbiosus* protocol. They completed a dedicated eligibility questionnaire, which updated and supplemented existing follow-up data on inclusion and exclusion criteria, and provided their informed consent. Exclusion criteria were: BMI below 18.5 kg/m^2^ or above 35 kg/m^2^, medical history of intestinal resection, organ transplant, type 1 diabetes, inflammatory bowel diseases, inflammatory or auto-immune diseases, cancer, chronic infectious diseases (e.g., HIV, hepatitis B or C) or chronic kidney disease, use of specific medication in the past 3 months (statins, antipsychotics, immunosuppressants, obesity-related treatment, antibiotics), a high habitual alcohol intake, a recent significant weight loss, current pregnancy or breastfeeding, current COVID-19 infection. Among participants who signed the consent form, a random selection was conducted to form 4 groups of 50 individuals, categorized based on their T2D status (yes/no, determined using self-reported information and health/medication follow-up data) and family history of T2D for non-diabetic participants (classified as: both parents, one parent, or none). BMI criteria were loosened for some participants to reach *n* = 50 per group: BMI below 18.5 kg/m^2^ (*n* = 2) or above 35 kg/m^2^ (35-40 kg/m^2^ : *n* = 8, 40-45 kg/m^2^, *n* = 4, > 45 kg/m^2^, *n* = 1). Selected participants were subsequently contacted by phone by the NutriNet-Santé coordination team to verify the exclusion criteria related to recent diagnoses, medication use or transient treatments (corticosteroids, non-steroid anti-inflammatory drugs, opioids, laxatives, proton pump inhibitors). Stool collection kits were then mailed to eligible participants. Each kit included a calibrated spoon (1 g) and a tube containing DNA/RNA Shield solution (Ozyme) to stabilize genetic material at room temperature for several days, following IHMS standards (https://mgps.eu/standard-operating-procedure/). Participants collected the samples themselves and returned them by mail to the SAMBO platform at INRAE MetaGenoPolis for further processing.

### Fecal DNA extraction and shotgun metagenomic sequencing

DNA extraction was carried out by the SAMBO platform at INRAE MetaGenoPolis, using MGP SOP 01 V1 (https://mgps.eu/sops/mgp-sop-001-v1/https://mgps.eu/sops/mgp-sop-001-v1/). Quality control and sequencing were performed by the MetaQuant platform at INRAE MetaGenoPolis with the protocol described in.^26^ For the construction of sequencing libraries, 500ng of high molecular weight DNA (> 10 kbp) were utilized. DNA preparation underwent quality control with FilterMax F3 (Molecular Devices, San José, US) and was assessed using DNA size profiling on a Fragment Analyzer instrument (Agilent Technologies, Santa Clara, US). DNA was sheared into fragments of approximately 150 bp using an ultrasonicator (Covaris, Woburn, US). The Ion Plus Fragment Library and Ion Xpress Barcode Adapters Kits (ThermoFisher Scientific, Waltham, US) were used for the DNA fragment library construction. The purified and amplified DNA fragment libraries were sequenced with the Ion Proton and Ion GeneStudio S5 Prime Sequencers (ThermoFisher Scientific, Waltham, US), generating at least 20 million high-quality 150 bp reads per sample on average. Quality control was conducted with AlienTrimmer:^27^ 1) sequencing adapters were removed; 2) low-quality reads were trimmed or discarded; and 3) reads shorter than 60 bp were discarded. Reads mapping to the human reference genome (NCBI RefSeq accession: GCF_009914755.1) were removed using bowtie2.^28^

### Generation of gene abundance table and taxonomic profiling

Using the METEOR software suite,^29^ the remaining high-quality reads were trimmed to 80 bases and aligned to two gene catalogs representing the human gut^30,31^ and human oral^32^ microbiota, respectively. Alignments with nucleotide identity lower than 95% were discarded, and gene counts were computed using a two-step procedure previously described for handling multi-mapped reads. The resulting raw gene count table was further processed using the R package MetaOMineR v1.31 (momr).^33^ Initially, it was downsizing to 20 million reads to account for variations in sequencing depth across samples. Subsequently, the rarefied gene counts were normalized by dividing by gene length, and a gene frequency table was generated using the FPKM normalization procedure.

### Taxonomic profiling with MetaGenomic Species

Using MSPminer,^34^ the gut and oral catalogs were previously organized into 1990 and 853 MetaGenomic Species (MSP), respectively.^31,32^ These MSP are clusters of co-abundant genes corresponding to the same microbial species. The taxonomic annotation of these MSP was determined using the Genome Taxonomy Database (GTDB) Release 08–RS214.^35^ The abundance of an MSP in a sample was defined as the mean abundance of its 100 marker genes (i.e., species-specific core genes that correlate the most together). If less than 10% of the marker genes were detected in a sample, the abundance of the MSP was considered null. MSP richness was assessed as the number of MSP detected in a sample (i.e., those with non-null abundance). Abundances at higher taxonomic ranks were computed as the sum of the MSP belonging to the given taxa. Taxonomic annotation was used to classify the different bacteria according to their Gram+ or Gram− wall composition. For MSP with unclassified taxonomy at species level, the Gram annotation was attributed on the basis of the status Gram of bacterial species belonging to the same phylum. Enterotypes were constructed, based on genus abundance, using the Dirichlet multinomial mixture models^36^ via R DirichletMultinomial package.^37^

### Predicted functional modules

Three databases were employed to predict gene functions: Kyoto Encyclopedia of Genes and Genomes (KEGG),^38^ eggNOG,^39^ and TIGRFAM.^40^ Genes from catalogs were mapped using DIAMOND^41^ onto KEGG orthologs (KO) from the KEGG database (version 8.9).

Each gene was assigned to the best-matching KEGG ortholog (KO) among hits with an e-value $<10e^{-05}$ and a bit score > 60. The same approach was applied to eggNOG (version 3.0). The gene catalog was also searched against TIGRFAM profiles (version 15.0) using HMMER 3.2.1.^42^ Subsequently, the presence of KEGG modules, gut metabolic modules (GMMs),^43^ and gut-brain modules (GBMs)^44^ in an MGS was assessed. A functional module is defined as a set of KOs (or NOGs, or TIGRFAMs). Since MGS represent pangenomes, their genes were categorized as either “core” genes (present in all samples containing the MGS) or “accessory” genes (which may be absent from a sample despite the detection of the MGS). Initially, a functional module was considered present in an MGS if at least 90% of its components were found among the core genes of the MGS. This assumption was then refined on a per-sample basis by including accessory genes detected in each specific sample. Finally, the potential of a module in a sample was quantified by summing the abundances of all MGS carrying that module within the sample. The catalogs were also annotated on the Carbohydrate-Active enZYmes Database (CAZy, November 2018) (http://www.cazy.org/).^45^ The abundance of each CAZYme group was computed using the same process as KEGG modules and GMM.^29^

### Network inference

Network inference was performed with OneNet-mean, a consensus network inference method that combines seven methods based on stability selection.^46^ Networks were inferred from the MSPs abundance table for each subgroup with a prevalence level filter at 50%, according to the parameter recommendations for the size of the dataset. The mean stability was fixed at 0.9 to ensure that each method produces a graph with similar density/precision and recall. The clustering was done with the CORE-Clustering method,^47^ with a frequency threshold of 70% for edge detection.

### Statistical analysis

The gradient effect of T2D risk on gut microbiota characteristics was investigated using a combination of approaches. First of all, rare species detected in less than 10% of samples were filtered out from the MSP abundance table. The non-parametric Wilcoxon-Mann-Whitney and Kruskal-Wallis tests were used to compare microbiota metrics across two or more conditions respectively. The effect size for these analyses was visualized using cliff delta. The non-parametric Spearman’s correlation test was used to examine the association between taxa abundance and numerical phenotype data. The effect size for these analyses was visualized via the correlation coefficient of the Spearman test. The non-parametric Fisher test was used to compare enterotypes across T2D risk groups. In our study, we considered the signal to be significant for a *p*value < 0.05 and an effect size > 0.2. The statistical tests were corrected using the Benjamini-Hochberg^48^ approach to control for multiple testing. All statistical analyses and visualizations were performed in the R environment version 4.4.1. Boxplots and barplots were generated using the R package ggpubr, and metagenomic analyses were conducted with the R package momr. Heatmaps were visualized using the ComplexHeatmap package. For alpha diversity analysis, the following metrics were used: observed richness (in intestinal or oral species) and the ratio of oral species to total species. For beta diversity analysis, Bray Curtis distances were calculated using the vegan package. The results from the univariate tests were validated through multi-adjusted models to account for the distribution of participant’s characteristics across T2D risk groups and adjust for potential confounding factors. The microbiota variable of interest was analyzed in relation to T2D risk group, adjusting for gender, age, BMI, IPAQ physical activity score, and smoking status (further referred to as covariates adjustment). To evaluate the significance of T2D risk group, we used the anova() function to compare models with and without T2D risk group, considering the T2D risk group’s contribution as significant if the p-value was below 0.05. For numeric microbiota variables (such as abundances and ratios), glm() models were applied, while multinom() models were used for categorical microbiota variables (e.g., enterotypes). The quality of the glm() models constructed was estimated by monitoring the normality of the residuals using the Jarque-Bera test and by the R square of the model.

Then we studied the potential impact of diet on the T2D risk-related gradient in microbiota features observed from the analyses described above. Dietary intakes were derived as a daily average across all available 24 h dietary records during the follow-up of participants prior to their stool sample collection, as an estimation of habitual intakes. Diet was studied as food group consumption, nutrient intakes and as dietary patterns. Dietary patterns were derived from a principal component analysis aiming to reduce the dimensionality of dietary data, to account for correlations between food groups while retaining most of the variation within the diet. The principal() function from the psych package with orthogonal varimax rotation was used on a data matrix including intakes of 28 food groups (g/day). The 3 retained factors (dietary patterns) were selected based on the scree plot, eigenvalues greater than 1.0, and interpretability criteria. Factor loadings indicated the correlation between dietary patterns and food groups. Loadings ≥ 0.30 or ≤ −0.30 were considered significant. The names of the dietary patterns were derived from the food groups with the highest loadings that characterized each pattern. Individual scores were computed to reflect the adherence of each participant to the derived patterns. Dietary intakes were studied along T2D status and family history. In parallel, we conducted analyses of associations between dietary variables and the microbiota features of interest, for which a gradient-like behavior was observed both in non-T2D only and in all participants. For this, models were constructed to explain microbiota variables as a function of T2D risk group and each dietary variable of interest added individually, along with covariates. The significance of T2D risk group and each dietary variable’s contribution was examined and considered at p<0.05. The glm() models were validated through visual inspection of residuals’ normal distribution, and the calc.relimp() function from the relaimpo package was used to calculate the relative importance of each variable in the model.

## Results

### Differences in participant’s characteristics in relation to T2D status and family history

A total of 192 participants with available metagenomic and dietary data were included in this study. In detail, 199 participants had submitted their metagenomic samples and 192 of these had valid dietary data. Participants were grouped according to their T2D status and their parental history of T2D: those with a T2D diagnosis (Group D, n = 46) and those without a T2D diagnosis with either no affected parents (Group 0, n = 47), one affected parent (Group 1, n = 48), or both affected parents (Group 2, n = 51). Group D demonstrated a significantly higher proportion of men, older age, higher BMI and lower educational level compared to the other groups. In addition, a gradient pattern was observed for BMI, physical activity and educational level across the 4 groups. Medication use at the time of stool sample collection was mostly observed in Group D, in line with their T2D status (see Table 1).

**Table 1.** Characteristics of participants according to T2D-risk group, NutriNet-Santé-homo symbiosus study, 2009–2021. Group 0 : non-T2D participants with 0 parents with T2D, Group 1 : non-T2D participants with 1 parents with T2D, Group2 : non-T2D participants with 2 parents with T2D, Group D : T2D-affected participants; *P-value obtained from Kruskal-Wallis and chi-square tests.

	Group 0 (*n* = 47)	Group 1 (*n* = 48)	Group 2 (*n* = 51)	Group D (*n* = 46)	P*
Sex (% W)	38 (80.9)	38 (79.2)	45 (88.2)	28 (60.9)	0.011
Age (median [IQR])	53.00 [48.00, 58.00]	53.00 [48.00, 54.25]	53.00 [48.00, 58.00]	58.00 [53.00, 63.00]	≤0.001
BMI (median [IQR])	23.24 [21.98, 24.87]	23.36 [21.71, 25.23]	24.68 [21.93, 27.75]	29.51 [24.04, 32.64]	≤0.001
Menopause (yes, %)	19 (50.0)	20 (52.6)	26 (57.8)	21 (75.0)	0.19
Smoking status (%)					0.63
Never smoker	26 (86.7)	26 (92.9)	25 (96.2)	16 (84.2)	
Former smoker	2 (6.7)	0 (0.0)	1 (3.8)	1 (5.3)	
Current smoker	2 (6.7)	2 (7.1)	0 (0.0)	2 (10.5)	
Physical activity score (%)					0.459
Low	17 (36.2)	9 (18.8)	13 (25.5)	14 (30.4)	
Medium	14 (29.8)	17 (35.4)	21 (41.2)	13 (28.3)	
High	16 (34.0)	22 (45.8)	17 (33.3)	19 (41.3)	
Educational level (%)					0.004
< 2 years of higher education	3 (6.4)	8 (16.7)	11 (21.6)	19 (41.3)	
≥ 2 years higher education	19 (40.4)	18 (37.5)	21 (41.2)	15 (32.6)	
≥ 5 years higher education	25 (53.2)	22 (45.8)	19 (37.3)	12 (26.1)	
Medication use					
NSAID (yes, %)	3 (6.4)	2 (4.3)	0 (0.0)	6 (13.3)	0.047
Antihypertensives (yes, %)	1 (2.1)	5 (10.6)	2 (4.0)	16 (35.6)	≤0.001
Proton pump inhibitors (yes, %)	0 (0.0)	1 (2.1)	2 (4.0)	6 (13.3)	0.015
Oral antidiabetics (yes, %)	0 (0.0)	0 (0.0)	0 (0.0)	31 (68.9)	≤0.001
Metformin (yes, %)	0 (0.0)	0 (0.0)	0 (0.0)	25 (55.6)	≤0.001

### Gradual differences in overall gut microbiota characteristics in relation to T2D status and family history

**Figure 1.**
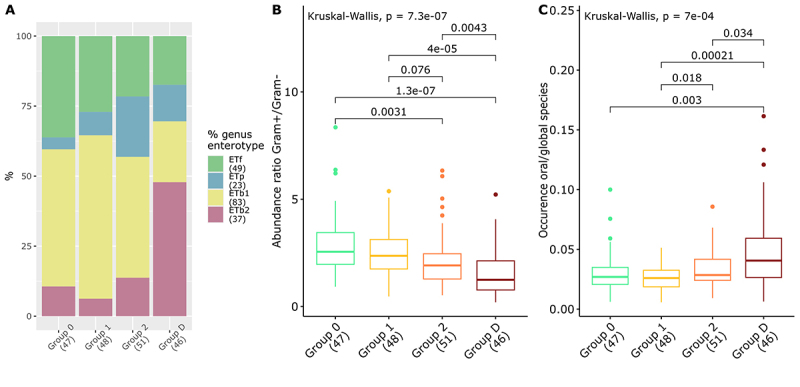
Gradient behaviour for overall microbiota metrics along groups of T2D status and family history. (A) Distribution of enterotypes calculated on genus level across groups. (B) Ratio of the abundance of Gram+ to Gram- bacterial species within the different groups. (C) Ratio of the number of species annotated as originating from the oral microbiota to total species richness within the different groups. Show P-values correspond to a Kruskal-Wallis test pairwise ones to a wilcoxon test. Only P-values ≤ 0.05 after correction are shown.

We first explored changes in overall gut microbiota characteristics as a function of increasing T2D risk, from Group 0 to Group D (see Figure 1).

Analysis using the enterotype approach revealed the existence of four enterotypes characterized by a majority of Firmicutes (EtF), Bacteroides1 (EtB1), Prevotella (EtP), and Bacteroides2 (EtB2), respectively. The distribution of enterotypes between groups revealed a non-significant progressive reduction (*p* = 0.19) in the proportion of individuals assigned to the EtF. There was a non-significant reduction from 36.2% in Group 0 to 21.6% in Group 2 (*p* = 0.12, Group 0 vs. 2) and 17.4% in Group D (*p* = 0.06 Group 0 vs. D). In parallel, the fraction of EtP individuals increased progressively (*p* = 0.05) from 4.3% in Group 0 to 8.33% in Group 1 and 21.6% in Group 2 (*p* = 0.02), but was 13% in Group D (*p* = 0.29, Group 2 vs. D). The fractions of samples annotated with EtB1 and EtB2 enterotypes were stable in Groups 0, 1 and 2 (mean (SD) = 50.1 (7.7)%, 10.2 (3.8) % respectively). However, in Group D, the EtB1 fraction was greatly reduced to 21.7% (*p* = 0.009, Group 0 vs. D) and the EtB2 fraction greatly increased to 47.8% (*p* = 8.5e-5, Group 0 vs. D) (Figure 1(a)). Covariates adjustment combined to alimentation score confirmed the associations between T2D risk group and the distribution of EtP (*p* = 0.004), EtB1 (*p* = 0.01) and EtB2 (*p* = 4.2e-5) enterotypes (see Figure 1(a)).

We further analyzed the ratio of Gram+ to Gram- species across the different groups and demonstrated a progressive and significant decrease (*p* = 7e-7) in the proportion of Gram+/Gram- bacterial species abundance from Group 0 to Group D (median (IQR) = 2.6 (2.0–3.4), 2.4 (1.8–3.1), 1.9 (1.3–2.5), and 1.2 (0.8–2.1), respectively) (see Figure 1(b)). Covariates adjustment confirmed the association between T2D risk group and the Gram+/Gram− ratio (*p* = 3e-4), although only a trend was observed when removing Group D (*p* = 0.07, Groups 0, 1, 2).

Looking at the invasion of the lower gut by oral species, another global marker of gut ecological status we observed a slight progressive and significant increase in the oral-to-gut species ratio from Group 0 and 1 to Group D (*p* = 0.59 for Group 0 vs 1; *p* = 0.037 for Group 1 vs 2 and *p* = 0.05 for Group 2 vs D), even considering the influence of proton pump inhibitor intake on this metric (see Figure S1). Covariates adjustment confirmed the association between T2D risk group and the oral-to-gut species ratio (*p* = 8e-4), although this association was no longer significant when removing Group D (*p* = 0.12) (see Figure 1(c)).

Overall, these observations suggested the existence of a trend toward a progressive evolution of microbiota in relation with family history of T2D and T2D status, even though the species richness of the gut microbiota was stable across the T2D risk groups (see Figure S2A).

### *Progressive development of an Enterocloster* species guild

We hypothesized that the shifts observed in gut microbiome composition along the T2D risk gradient linked with family history could reflect changes in the interactions between bacteria within the gut ecosystem. Therefore, we aimed to investigate the association of T2D status and family history with microbial guilds, defined as groups of co-abundant species inhabiting similar ecological niches within an ecosystem.

**Figure 2.**
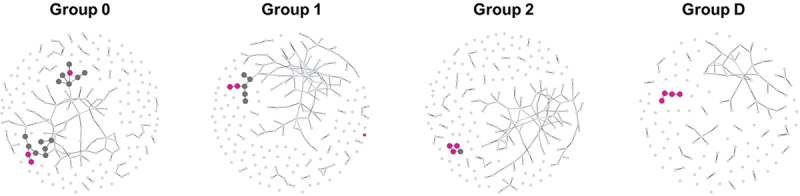
Network graphs with a focus on microbial guilds containing *enterocloster* species. The clustering step revealed around ten guilds for each group studied but only guilds containing at last one species of the *enterocloster* genus were highlighted with a larger node size. *enterocloster* species are shown in fuchsia.

We inferred and clustered a network for each group, and then employed a visual analysis to explore changes relative to T2D status and family history. Among the different guilds detected, we identified the *Enterocloster* species guild for which a gradient-like behavior was observed, with an increased number of *Enterocloster* species linked together within the same guild (see Figure 2). Indeed, in Group 0, *Enterocloster* species were dispersed between two different guilds. Then, their number gradually increased, moving from two species within the same guild in Group 1 to three in Group 2 before clustering into an *Enterocloster*-specific guild with four species in Group D. The species of the *Enterocloster* genus are often linked to adverse conditions. These four species (*E. aldenensis, E. asparagiformis, E. bolteae, E. citroniae*) included in the graphs were significantly more abundant in Group D than in all other groups (see supplementary table). Note that *E. aldenensis* is absent from networks 0, 1 and 2 because its prevalence is below the threshold of 50% in these groups.

### Species and functional modules following a gradient-type behaviour along T2D status and family history

Our study then involved further exploration of gradient-type behavior in species and functional potential. To do so, we identified markers for which consistent associations with an increasing T2D risk was observed considering 1) T2D family history gradient in non-T2D individuals (Group 0–1–2), 2) T2D family history gradient and status in all individuals (Group 0–1–2-D).

**Figure 3.**
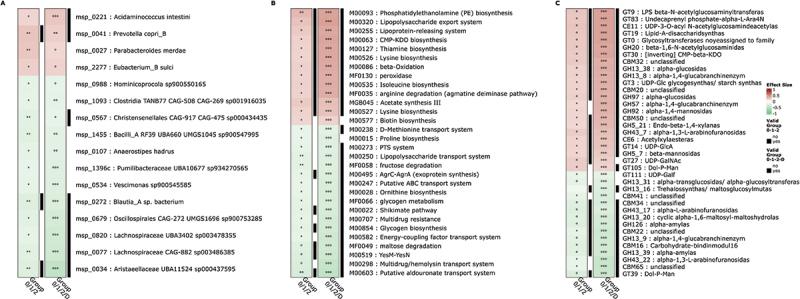
Heatmap of species (A), functional modules (B) and CAZymes (C) whose abundance is associated with a T2D risk gradient linked with family history. Focus on items exhibiting a significant association in both analyses with and without T2D participants (p-value ≤ 0.05 and effect size ≥ 0.2). Information on significance (*p value ≤ 0.05, **pvalue ≤ 0.01, ***p value ≤ 0.001, °*q* value ≤ 0.3, °°*q* value ≤ 0.1, °°°*q* value ≤ 0.05). Colors indicate the direction (red: gradient in favour of T2D, green: gradient in favour of non-T2D) and the rho value of the Spearman correlation test of the associations. Indication of validation after covariates adjustment: the association with T2D risk group remained significant (black) or not (white).

Based on these criteria, the analyses identified 16 MSP (MetaGenomic Species) of interest, 12 with an abundance gradient in favor of the non-T2D groups and 4 with an abundance gradient in favor of the T2D group. Among the 16 MSP of interest, 5 have been isolated. Among then, 1 (*Anaerostipes hadrus*) is negatively associated with the T2D risk gradient whereas the 4 others (*Prevotella copri*, *Parabacteroides merdae*, *Eubacterium sulci* and *Acidaminococcus intestini*) have abundance gradients in favor of T2D and non-T2D groups with antecedents. However, after covariates adjustment, associations remained significant only for *P.copri* (msp0041), *Aristaeellaceae sp* (msp0034) and *Blautia_A sp*. (msp0272) (see Figure 3(a)).

Similarly, 30 functional modules were selected, 13 were enriched in the non-T2D groups, including modules involved in glycogen, chorismate, proline and ornithine metabolism, and 17 were enriched in the T2D group, including modules involved in lysine, biotin, acetate, isoleucine, CMP-KDO (CTP:CMP-3-deoxy-manno-octulosonate cytidylyltransferase), peroxidase and thiamine metabolism, as well as in arginine degradation. After covariates adjustment, only 6 modules abundances trend remained significantly enriched in non-T2D groups and 12 in T2D group (see Figure 3(b)).

Finally, 38 CAZymes exhibited a gradient-like behavior. Of these, 13 showed a gradient in favor of non-T2D groups, including alpha-L-arabinofuranosidase, Trealossynthas and 1,4-alpha-glucan branching enzymes, and 25 in favor of the T2D group, including LPS N-acetylglucosaminyltransferase, Lipid-A-disaccharide synthase and LpxC, among others. After covariates adjustment, associations were confirmed for 11 CAZymes in favor of non-T2D and 20 in favor of T2D (see Figure 3(c)).

### Dietary intakes related to gradients in T2D risk and gut microbiota

Three dietary patterns were retained. Dietary pattern 1 included “high simple carbohydrate” food groups, with positive loadings (≥ 0.35) for sweets products, pastries, sweet biscuits, and sweet nonalcoholic beverages. Dietary pattern 2 included “high fiber” food groups, with positive loadings (≥ 0.35) for vegetables, oilseeds, whole grains, legumes, fruits, unsweetened nonalcoholic beverages, and soup. Finally, dietary pattern 3 included “high animal protein” food groups, with positive loadings (≥ 0.35) for processed meat, red meat, added fat, tuber, refined cereals, eggs, alcoholic beverages, cheese. (see Table S1)

**Figure 4.**
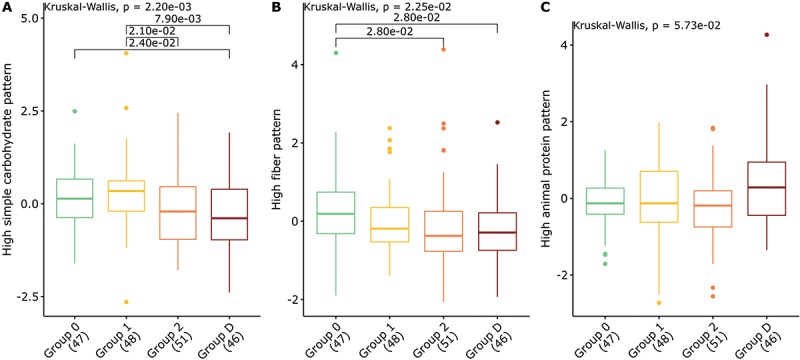
Differences in dietary pattern scores between groups for (A) the ‘high simple carbohydrate’ pattern, (B) the ‘high fiber’ pattern and (C) the ‘high protein’ pattern. A higher dietary pattern score indicates a higher adherence to this pattern. Show P-values correspond to a Kruskal-Wallis test pairwise ones to a wilcoxon test. Only P-values ≤ 0.05 after correction are shown.

Group 2 and Group D showed significantly lower scores on the “high fiber” dietary pattern compared to Group 0. Group 2 and Group D also exhibited lower scores on the “high simple carbohydrate” dietary pattern compared to Group 0 and Group 1. Finally, scores on the “high animal protein” dietary pattern were similar for the 3 non-T2D groups, but higher in Group D. (see Figure 4)

**Figure 5.**
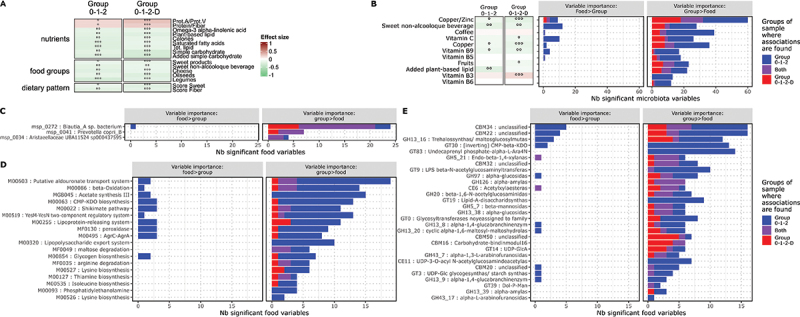
Associations between diet and gut microbiota features. (A) Heatmap of dietary variables exhibiting a gradient-like behaviour along T2D risk linked with family history. Focus on items with significant associations in both analyses with and without T2D participants (p-value ≤ 0.05 and an effect size ≥ 0.2). Information on significance (*p value ≤ 0.05, **p value ≤ 0.01, ***p value ≤ 0.001, °*q* value ≤ 0.3, °°*q* value ≤ 0.1, °°°: qvalue ≤ 0.05). Colors indicate the direction (red: gradient in favour of T2D, green: gradient in favour of non-T2D) and the rho value of the Spearman correlation test of the associations. (B) Bar plot of the number of microbiota variables of interest significantly influenced by dietary variables. Distinction between cases where the weight of the influence of dietary variables is greater than that of the group (left) and cases where the weight of the influence of dietary variables is less than that of the group (right). Distinction of associations between dietary variables and microbiota features found in Group 0–1–2 analyses only (blue), in Group 0–1–2-D analyses only (red) or in both analyses (purple). Focus on the 11 dietary variables with the highest number of associations with microbiota variables. Information about gradient-like behaviour along T2D risk via heatmap. Colors indicate the direction (red: Gradient in favour of T2D, Green: gradient in favour of non-T2D) and the rho value of the Spearman correlation test of the associations. (C-E) barplot of the number of dietary variables significantly associated with the different microbiota variables of interest (species: (C) functional modules: (D) CAZymes: E)). Distinction between cases where the weight of the influence of dietary variables is greater than that of the group (left) and cases where the weight of the influence of dietary variables is less than that of the group (right). Distinction of associations between dietary variables and microbiota features found in Group 0–1–2 analyses only (blue), in Group 0–1–2-D analyses only (red) or in analyses (purple).

Using the same approach used to identify microbiota components of interest, 16 dietary variables exhibited gradient-like behavior as a function of T2D status and family history. Two of these showed a gradient in favor of T2D (protein/fiber ratio and a trend for animal protein/vegetable protein ratio), while 14 showed a gradient in favor of no T2D (4 nutrients, 8 food groups and 2 dietary patterns), see Figure 5(a).

At the same time, the identification, using linear models, of the dietary variables significantly influencing the components of interest in the microbiota revealed several pieces of information. Firstly, the 10 dietary variables influencing the greatest number of distinct components of interest in the microbiota included, at nutrient level, vitamins A, B9 and B5, C, simple carbohydrates, copper and the copper/zinc ratio, and, at food level, sweet drinks, fruit, coffee and water. For all these dietary variables, the number of unique components of the microbiota influenced was greater when the analyses were carried out only on the groups without T2D (Group 0–1–2) than when the group of T2D volunteers was taken into account (Group 0–1–2-D) (see Figure 5(b)). It is also interesting to note that of these 10 most influential dietary variables, only sweetened beverage and simple carbohydrate intakes were identified as having gradient-type behavior (see Figure 5(a)). Furthermore, analysis of the dietary variables significantly influencing the abundance of each component of interest in the microbiota revealed marked differences between these components. For instance, Blautia_A sp. was influenced by 19 and 21 dietary variables in Group 0–1–2 and Group 0–1–2-D while *P.copri* and *Aristaeellaceae sp*. were influenced by less than 10 food components. Analyses of functional modules and CAZymes revealed a different behavior than that observed for species, with a greater number of associated food variables for Group 0–1–2 compared with Group 0–1–2-D and a much lower proportion of combined signals. Functional modules and CAZymes were associated on average with 10 (range : 2–24) dietary variables. Among these, some were strongly associated with a large number of dietary items, such as beta-oxidation (M00086) (n = 13), acetate synthesis (MGB045) (n = 15) or CMP-KDO (M00063) (n = 12) modules, as well as trehalo-synthetase (GH13_16) (n = 9) or LPS construction (GT9) (n = 10) CAZymes. Conversely, there were items with very few associations with dietary variables, such as the lysine (M00526) (n = 2), thiamine (M00127) (n = 4) or isoleucine (M00535) (n = 4) production modules. (see Figure 5(c)).

## Discussion

Our study highlighted significant shifts in the composition of the gut microbiota associated with increasing T2D risk according to family history, particularly in individuals without T2D but with 2 T2D-affected parents compared to 0. These microbiota changes were observed through proxy of the ecoystem like enterotypes, as well as more specific characteristics such as species composition and functional potential. By adjusting for covariates and dietary factors, we were able to identify microbiota markers that were specifically related with family history, independently from diet. These findings underline the potential role of the gut microbiota potential as both an indicator of T2D risk and an active factor in the disease’s development and progression.

### Ecosystemic and compositional microbiota level shifts

Our findings revealed that non-T2D individuals with no or a single T2D-affected parent displayed highly similar microbiota compositions, while T2D individuals had a distinct microbiota profile. Notably, non-T2D individuals with two T2D-affected parents showed a microbiota composition that differed from both T2D individuals and non-T2D individuals with one or no parent with T2D. These differences were apparent in the overall microbiota composition visualized using PCoA and in enterotypes (see Figure S5). The enterotype-based approach provided a robust and interpretable perspective on microbiota changes associated with T2D risk, offering categorical insights that enhanced the detection of the risk gradient. While less granular than PCoA, its reduced sensitivity to inter-individual variability made it a valuable complement to the continuous, high-resolution view offered by ordination-based methods. It highlighted trends such as a slight, non-significant reduction in “Firmicutes” enterotype and an increase in “Prevotella” enterotype among non-T2D individuals with a growing family history-related T2D risk. Additionally, it revealed a decrease in “Bacteroides1” enterotype and an increase in “Bacteroides2” enterotype specifically in individuals with T2D.

The trends observed in the “Firmicutes” and “Bacteroides2” enterotypes are consistent with the results of other studies at the phyla level showing a decrease in Firmicutes and an increase in the Bacteroidetes phyla in diabetics compared with people without T2DM.^49,50^ Importantly, these changes were not related to dietary influences like decreased fiber or increased carbohydrate intake (see Figure S3), which are known to affect the “Firmicutes” and “Bacteroides” enterotypes, respectively.^51^

Additionally, the gradual decrease in the Gram+/Gram- ratio with family history is consistent with existing literature on microbiota composition^52,53^ and systemic LPS levels (a pro-inflammatory component of Gram- bacteria).^54^ The current hypothesis regarding LPS suggests that its increase within the gut lumen may elevate digestive inflammation^55^ and potentially compromise the gut epithelial barrier.^56^ This permeability of the barrier can lead to metabolic endotoxemia which allows LPS to enter the circulation, activating inflammatory pathways (CD14/TLR4) which, when maintained, can lead to beta cell death in the pancreas, straining surviving cells and ultimately increasing insulin resistance.^14,54^ This enrichment of LPS in T2D can also be seen indirectly by monitoring the ratio of Firmicutes/Bacteroidetes phyla (see Figure S2B), which is consistent in our study with the Gram+/Gram- gradient and with previous studies.^50^

In addition to LPS-driven inflammation, the increase of oral microbiota species proportion in the gut may contribute to intestinal inflammation in T2D in correlation with periodontal diseases.^57^ Indeed, a bidirectional link exists between T2D and periodontal disease:^58^ oral inflammation and microbiota translocation can promote intestinal inflammation,^59^ while T2D-related IL-17 production may influence the oral microbiota,^60^ heightening periodontal disease risk. However, information regarding periodontal disease diagnosis was not available in our cohort. Furthermore, because this was a cross-sectional analysis, we cannot conclude whether the increased prevalence of oral species in T2D patients reflects an effect of the disease rather than a cause. However, the low presence of this signal among non-T2D individuals further supports the “effect” hypothesis, even though a significant increase in oral species was also detected in non-T2D individuals with 2 T2D-affected parents.

Among the other enterotypes, while the “Bacteroides1” enterotype has not been associated with T2D in the literature, the “Prevotella” enterotype, and more specifically *P.copri*, was associated in Western populations with oral microbiota^61^ and pro-inflammatory phenotypes such as obesity.^62^ In our cohort, a possible translocation of oral to intestinal microbiota may account for the elevated signal for “Prevotella” enterotype in T2D individuals.

Overall our observations further support the concept of a close inter-connection between the gut microbiota and host parameters, including gut barrier integrity, the immune status and oxidative stress.^63^

### Microbial taxonomic and functional alterations

Detailed microbiota metrics underscored microbiota alterations that may contribute to T2D development. These included an increased abundance along T2D risk gradient for specific functional modules and CAZymes (M00320, M00255, M00063, GT9, GT83, CE11, GT30) involved in LPS synthesis, as well as for modules (M00526, M00535, M00527) responsible for producing BCAAs like leucine and isoleucine, which repress lipolysis and promote insulin resistance,^13^ or else for some CAZymes (GH13_8, GH97, GH20) having alpha-amylase activity, breaking down starch into glucose and triggering an increased insulin response.^64^

Beyond these modules with potentially harmful activities, other pathways were enriched, involved in the synthesis of elements observed to be depleted in the bloodstream of T2D patients, including thiamine (M00127)^65^ and agmatine (MF0035).^66^ The abundance gradient of these pathways along increased T2D risk linked with family history may reflect host-microbiota communication, with microbiota compensating for the host’s deficiencies (e.g., reduced intestinal absorption and/or host production) – a mechanism previously documented for biotin^67,68^ but not for thiamine and agmatine. Further research could clarify similar mechanisms for these last 2 nutrients. Among the pathways depleted along the T2D risk gradient, we identified CAZymes and modules that convert glucose to glycogen (M00854) or isomaltulose (GH13_31) and may therefore help regulate blood sugar levels by either storing glucose within the microbiota^69^ or reducing beta-cell response,^70^ potentially leading to a lower risk of insulin resistance. These CAZymes (GH13_16) can also convert maltose into trehalose, whose beneficial effects on CRP-dependent inflammation in T2D^71^ could be mediated by a reduction in the inflammatory activity of macrophages in response to microbiota-derived LPS stress.^72^

Associations with T2D risk gradient linked with family history were also observed at the species level, including *P. copri*, *Aristaeellaceae.sp* and *Blautia_A sp*. However, only *P.copri* is referenced in the literature for its direct association with T2D via the production of branched-chain amino acids.^73^ In addition, an increase in the abundance of the four *Enterocloster* species has already been associated with T2D in the literature.^9^ These bacteria also produce ethanol^74^ and trimethylamine (TMA), the latter molecule being associated with red meat intake^75^ and with a higher risk of T2D.^76^ This is consistent with the observed gradient toward a higher ratio of animal to plant protein intake along T2D risk and a higher adherence to a “high animal protein” dietary pattern in T2D individuals.

Examining the functional potential of these species underlined possible significant contributions of *P. copri* and *Aristaeellaceae.sp* in T2D development, with *P. copri* driving activity in alpha-amylase, BCAA, and LPS production, and *Aristaeellaceae.sp* contributing to glucose-to-glycogen conversion and ornithine synthesis. These findings support the hypothesis that microbiota alterations associated with increasing T2D risk linked with family history may favor an LPS-mediated inflammatory environment and suggest that the microbiota adapts to the host’s health status by modulating production of critical metabolites. The concentration of these functional activities in two species highlights them as potential candidates for future intervention studies.

### Diet and microbiota

The observed microbiota signals suggested associations between microbiota alterations and a T2D risk gradient. However, such associations could be confounded by diet since diet has an impact on both T2D (and, conversely an individual's diet may be impacted by T2D status), and microbiota composition. Our study highlighted differences in dietary intakes across T2D risk groups. Notably, both diabetic and non-diabetic individuals with a family history of T2D exhibited reduced intake of simple carbohydrates (naturally present and added sugars), and lower overall caloric intake compared to non diabetic individuals without a family history of T2D. These results may reflect the adherence of T2D individuals and T2D-predisposed individuals to T2D preventive dietary guidelines, targeting dietary intakes known to be associated with an increased risk of T2D, influenced by parental dietary habits and/or medical advice.^77–79^ However, such dietary pattern was also accompanied by a reduction in the consumption of fiber-rich foods, while dietary fibers are beneficial for the prevention and management of diabetes,^80–82^ which was also reflected in a higher protein-to-fiber ratio, documented to have a detrimental impact on the gut microbiota.^83^ An increase in the “high animal protein” pattern (also characterized by the consumption of refined cereals) was also observed in T2D. Overall, this highlights the importance of dietary advice in T2D, which should not only focus on simple carbohydrates but aim at a balanced diet, notably promoting dietary fibers, in line with the most recent guidelines.^84^ Among the list of dietary variables with an association with the T2D risk gradient, only a fraction had a significant influence on the markers of interest of the microbiota with gradient-type behavior. The main dietary metrics influencing these microbiota markers do not therefore exhibit gradient-type behavior. This dichotomy could illustrate the influence of host metabolism, which can act as a bridge between diet and microbiota. Also, associations between diet and gut microbiota features were especially apparent among non-diabetic individuals with or without a family history of T2D, suggesting that while diet may significantly shape microbiota in the absence of disease, its relative influence may be lessened once T2D is established.

Specific dietary components showed a marked effect on microbiota function. For instance, vitamin C, which supports gut barrier function and reduces inflammation,^85^ and is closely associated with fiber intake, as well as sugar-sweetened beverages, were associated with microbiota functions involved in fiber degradation and sugar metabolism (e.g., MF0066, CBM65, GH43_22, CBM22, GH43_17, GH13_9). The copper/zinc ratio displayed a distinct pattern, with associations observed in both non-diabetic and diabetic individuals with microbiota features for which a gradient-like behavior was highlighted. This copper/zinc dietary intake ratio has been previously associated with T2D risk, showing an inverse association for a ratio below 0.5 and a positive association for a ratio above 0.5.^86^ This shift in association may stem from interactions between the gut microbiota and the immune system: zinc generally reduces intestinal inflammation, fostering bacterial growth, whereas copper has antibacterial properties by activating immune responses.^87^ Additionally, copper-driven inflammation is a known contributor to T2D development.^88^ One possible explanation is that a high copper-to-zinc ratio could increase copper-dependent inflammation related to T2D. Consequently, in our cohort, with a copper/zinc ratio below the threshold of 0.5, the low inflammation effect of copper may potentially protect from pathogenic bacteria that persist once T2D is established. Interestingly, some microbiota markers, such as *P. copri*, *Aristaeellaceae.sp*, and BCAA production modules, showed almost no association with diet.

Finally, it should be noted that all associations between gut microbiota features and T2D risk gradient linked with family history remained statistically significant even after adjustment for diet or covariates variables, suggesting complementary (although sometimes partly interrelated) effects. As a result, we can envisage the existence of a specific link between the markers of interest in the microbiota and the T2D risk gradient.

### Strengths and limitations

Our study had several strengths including an original approach by monitoring the number of parents affected by type 2 diabetes (T2D) to assess familial risk. Also, the metagenomic analysis of gut microbiota provided a detailed view of its functional potential. Third, the availability of detailed long-term dietary data allowed us to establish connections between diet and gut microbiota. However, some limitations should be acknowledged like the non-T2D group with two T2D-affected parents might include individuals with undiagnosed T2D or pre-diabetes, and our hypotheses regarding increased LPS production, gut permeability, and inflammation could be further explored through blood-based markers like LPS, CRP, zonulin, BCAAs, and glucose. Also, many microbiota-related signals were lost after statistical corrections or accounting for covariates factors, highlighting the need for larger sample sizes. Finally, dietary effects such as fiber intake appear significant only above certain thresholds (30 g/day for fibers^89^), emphasizing the need for interventional studies to confirm these links.

## Conclusions

Our study identified a gradient in microbiota composition associated with family history (number of affected parents) and T2D status. This gradient was marked by an increase in bacterial populations producing LPS and branched-chain amino acids (BCAAs), coupled with a reduction in bacteria that convert glucose to glycogen; a non-insulin stimulating form and possess anti-inflammatory properties. Moreover, the findings suggested that diet influenced microbiota alterations, with potential contributions from red meat, sugar-sweetened beverages, vitamin C, copper, or zinc. However, dietary and covariates factors alone did not fully account for the microbiota gradient linked to T2D risk. In summary, our study highlights microbiota patterns shaped by T2D risk linked to family history and by dietary factors. These insights, which should be confirmed by mechanistic approaches, may contribute to our understanding of T2D development through the gut microbiota and may guide future efforts in early T2D diagnosis and the therapeutic use of microbiota in disease management.

## Supplementary Material

Supplemental Material

## Data Availability

Raw data described in the manuscript are protected and are not available due to data privacy laws according to French regulations. Both metagenomic and participant’s data can be made available upon request pending application and approval. Researchers from public institutions can submit a request to have access to the data for strict reproducibility analysis (systematically accepted) or for a new collaboration by providing information about their institution and a brief description of the project to collaboration@etude-nutrinet-sante.fr. All requests will be reviewed by the steering committee of the NutriNet-Santé study. If the collaboration is approved, a data access agreement will be required, and any necessary authorizations from the relevant administrative authorities may be needed. In compliance with existing regulations, no personally identifiable data will be accessible.
