# Supplementary material for: Alterations in gut microbiota characteristics along a type 2 diabetes risk gradient linked with family history
Source: Gut Microbes Rep. 2025 Jul 22;2(1):2527766. doi: 10.1080/29933935.2025.2527766 (PMC12940137; doi:10.1080/29933935.2025.2527766)
Supplement: Supplemental Material [file KGMR_A_2527766_SM7523.docx]

| 1098 | **Supplementary Material** |
| --- | --- |


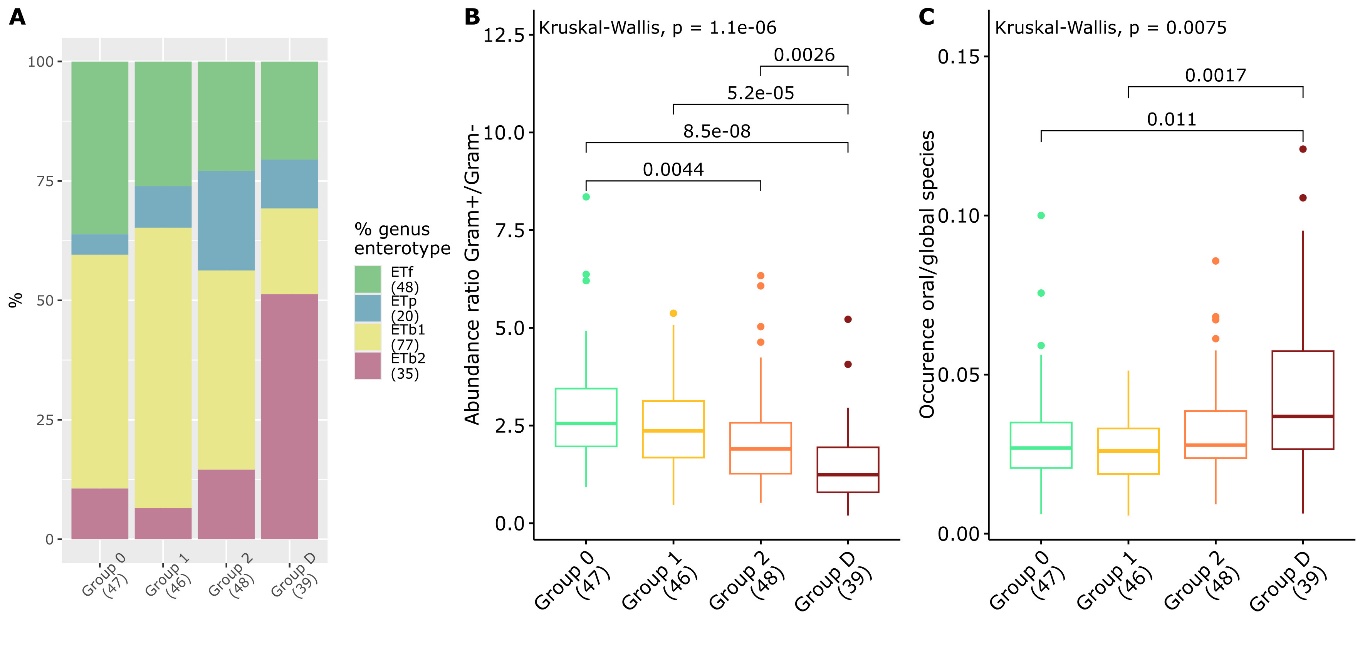


**Fig. S1**: Gradient behaviour for overall microbiota metrics along groups of T2D status and family history without proton pump inhibitor.

1. Distribution of enterotypes calculated on genus level across groups. B) Ratio of the abundance of Gram+ to Gram- bacterial species within the different groups. C) Ratio of the number of species annotated as originating from the oral microbiota to total species richness within the different groups. P-values were derived from Wilcoxon and Kruskal-Wallis tests


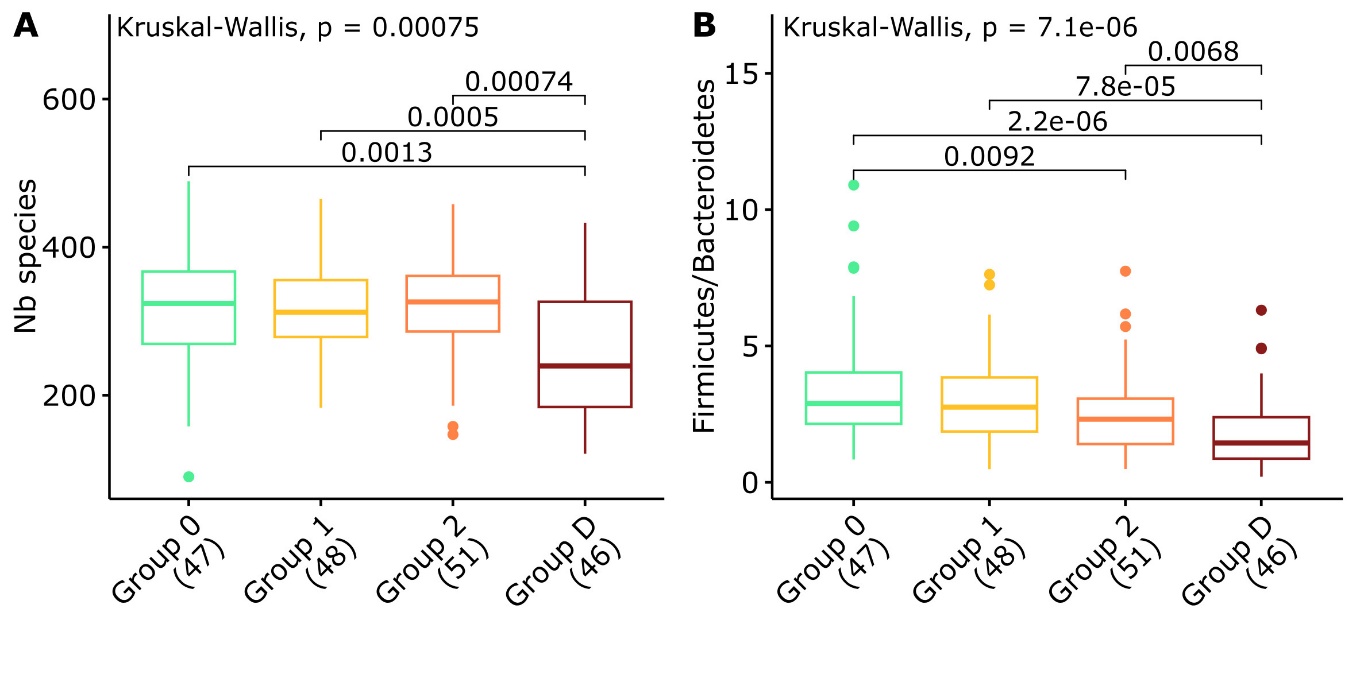


**Fig. S2**: Gradient behaviour for A) species nomber or microbiota richness and B) Firmicutes/bacteroidetes ratio along groups of T2D status and family history. P-values were derived from Wilcoxon and Kruskal-Wallis tests


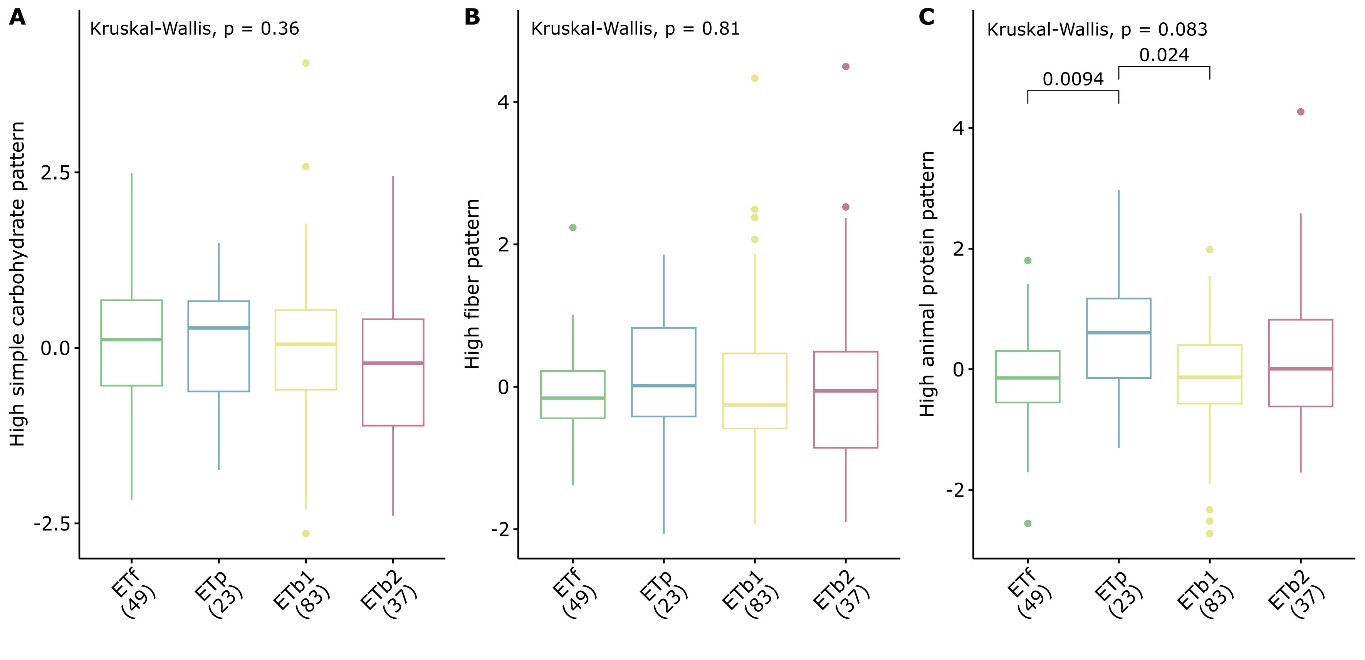


**Fig. S3**: Differences in dietary pattern scores between enterotypes for A) the ’High simple carbohydrate’ pattern, B) the ’High fiber’ pattern and C) the ’High protein’ pattern. A higher dietary pattern score indicates a higher adherence to this pattern. P-values were derived from Wilcoxon tests. All study samples were used


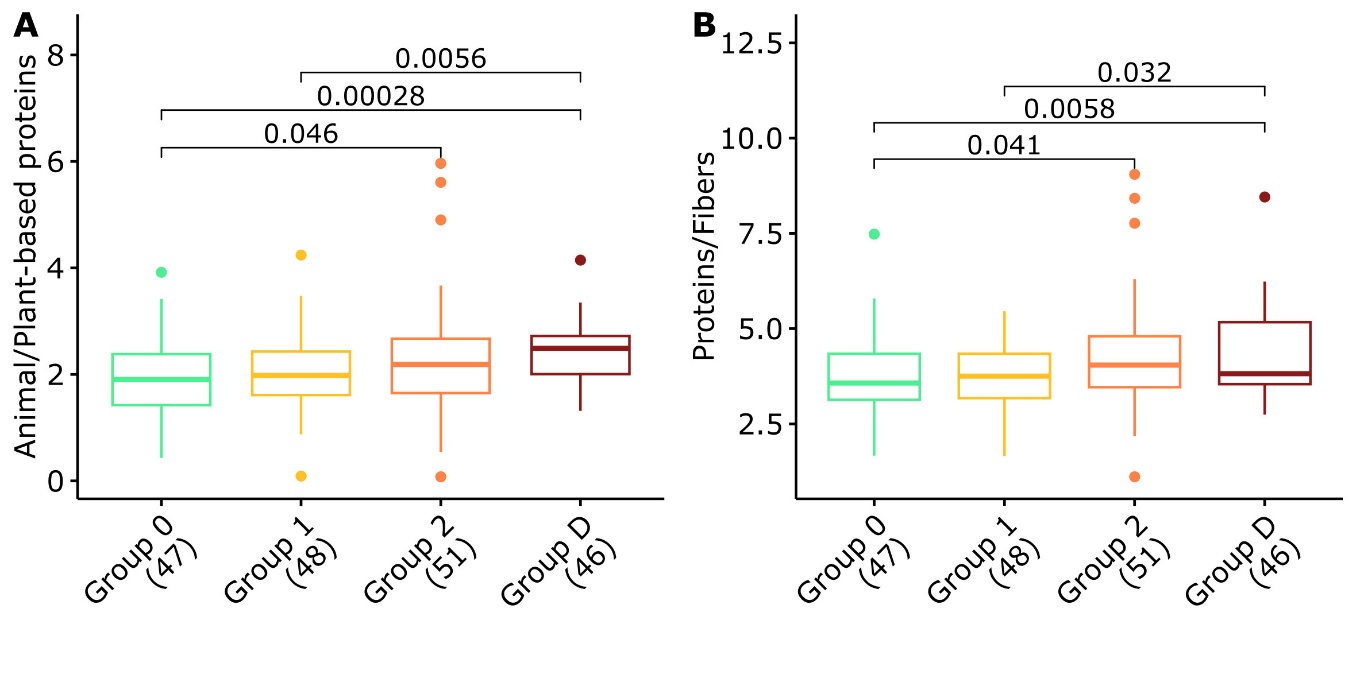


**Fig. S4**: Gradient behaviour for A) animal/plant-based protein ratio consumed and

1. protein/fiber ratio consumed along groups of T2D status and family history. P- values were derived from Wilcoxon and Kruskal-Wallis tests


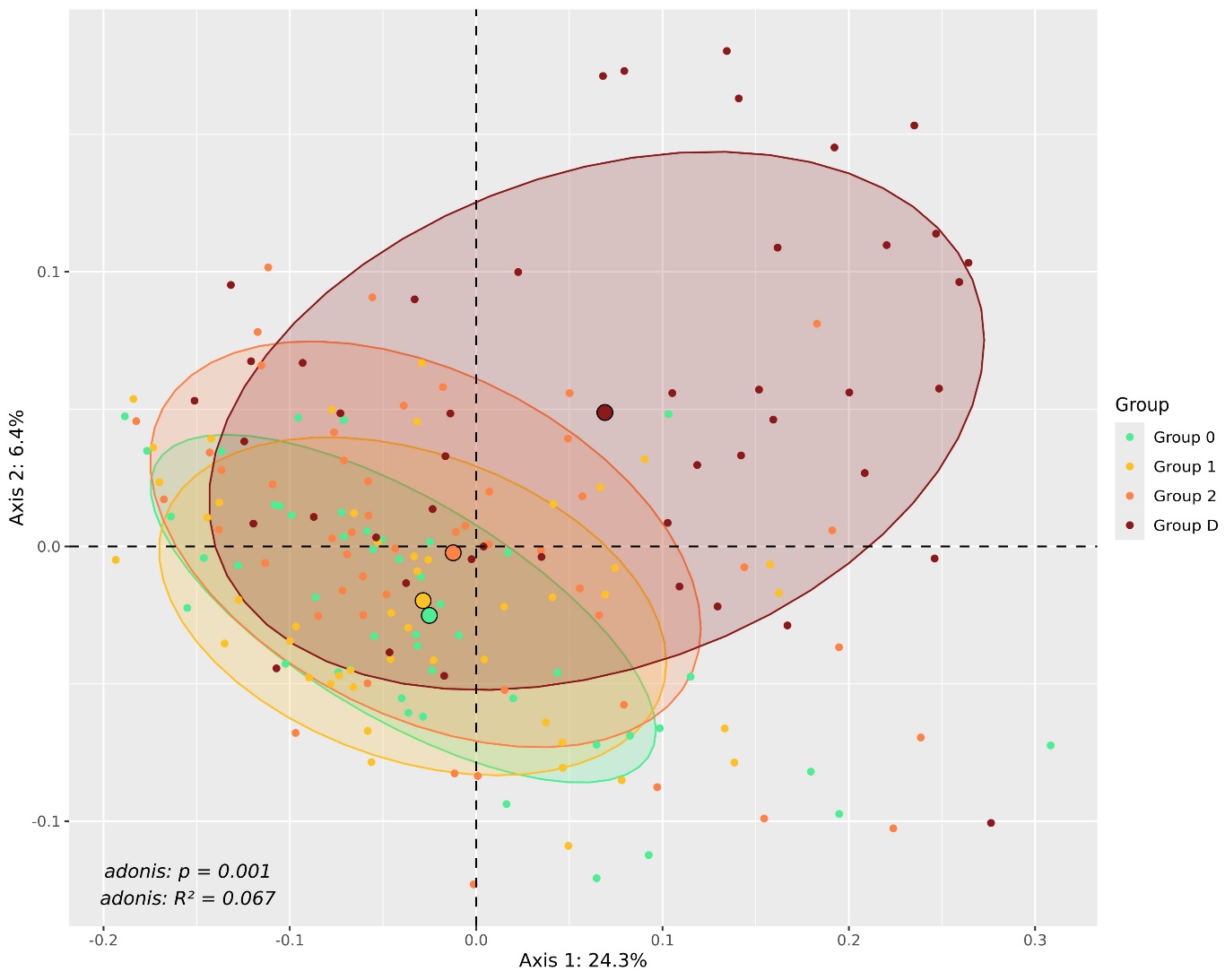


**Fig. S5**: Principal Coordinates Analysis (PCoA) based on the Bray-Curtis dissimi- larity matrix at family-level of all human fecal metagenomes (n=192). Each small dot represents a sample, large dots are groups centroids. Comparisons were carried out with PERMANOVA tests. Coloring and tests according to antecedants status.


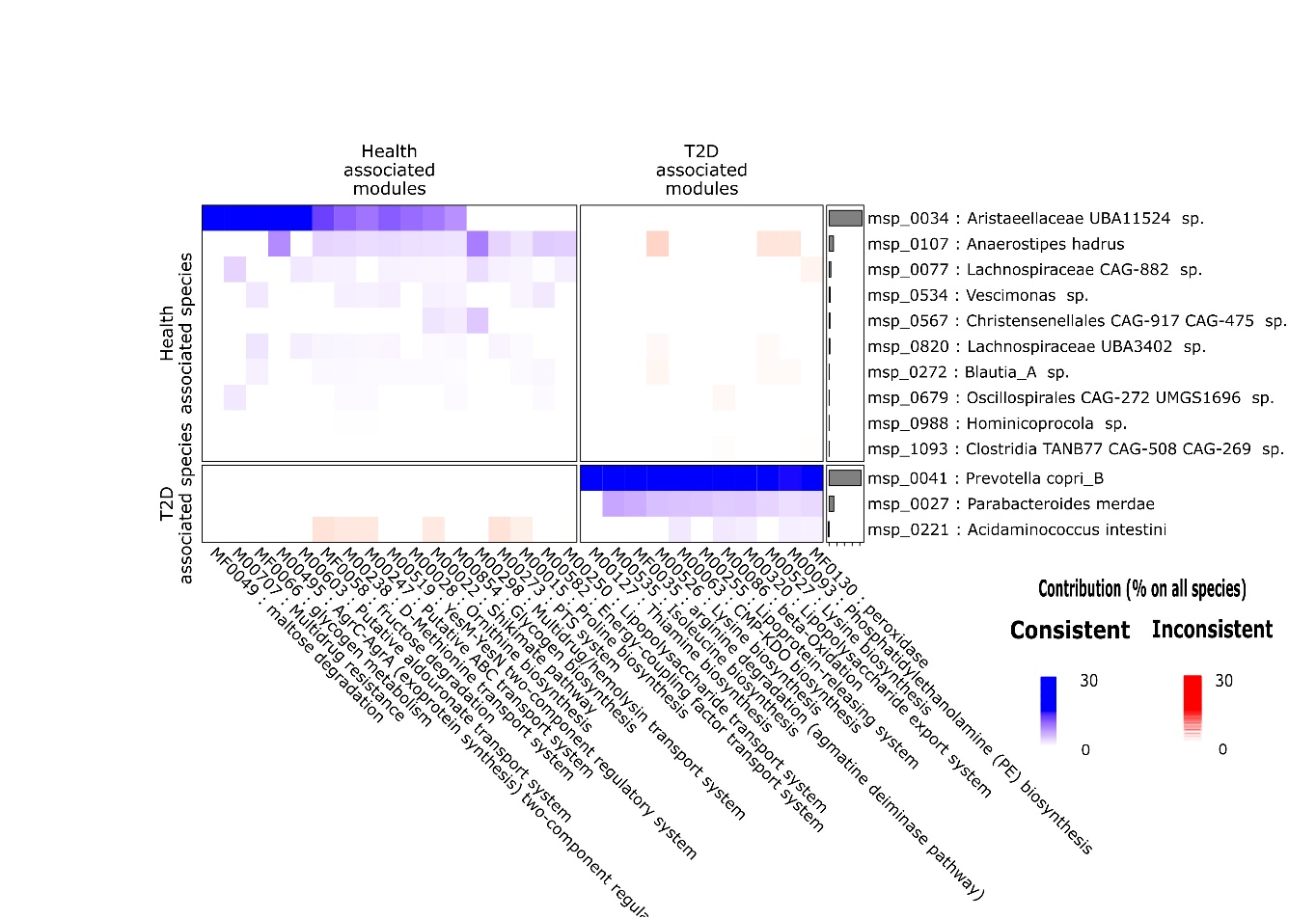


**Fig. S6**: Contribution of species of interest to the abundance of functional modules of interest. Heatmap of the mean percentage abundance of the functional module explained by the species for all samples in the cohort (n=192). Indication of whether the species contributes to a functional module with equivalent behaviour according to family history (blue) or inconsistent behaviour (red).


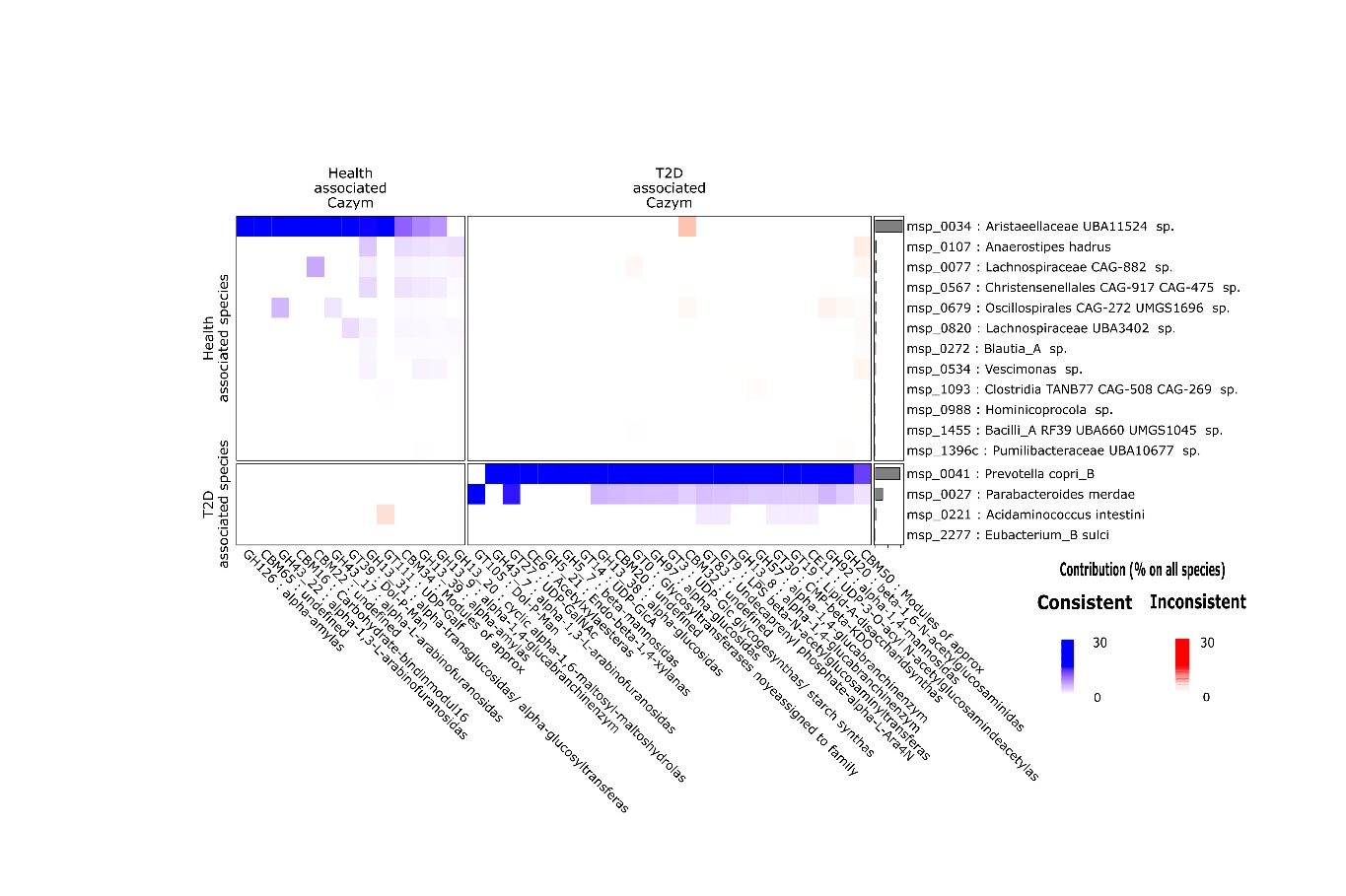


**Fig. S7**: Contribution of species of interest to the abundance of CAZymes of inter- est. Heatmap of the mean of the percentage of CAZymes abundance explained by the species for all samples in the cohort (n=192). Indication of whether the species con- tributes to a CAZyme with equivalent behaviour according to family history (blue) or inconsistent behaviour (red).

**Table S1**: Table of 25 food group scores for each food pattern

|  | Pattern 1 | Pattern 2 | Pattern 3 |
| --- | --- | --- | --- |
| Sweet products | 0.703 | -0.048 | 0.138 |
| Pastries | 0.585 | 0.045 | -0.008 |
| Sweet biscuits | 0.502 | -0.111 | 0.150 |
| Sweet non-alcoholic beverages | 0.398 | -0.033 | 0.004 |
| Breakfast cereal | 0.275 | 0.089 | -0.224 |
| Dairy products | 0.095 | -0.198 | -0.147 |
| Vegetables | -0.224 | 0.727 | 0.136 |
| Oilseeds | 0.152 | 0.653 | -0.056 |
| Whole grains | -0.127 | 0.586 | -0.031 |
| Legumes | 0.195 | 0.584 | 0.008 |
| Fruits | 0.174 | 0.550 | -0.030 |
| Unsweetened non-alcoholic beverages | -0.038 | 0.454 | 0.040 |
| Soup | -0.243 | 0.437 | 0.140 |
| Cold cuts | 0.015 | -0.200 | 0.687 |
| Meat | -0.091 | -0.324 | 0.654 |
| Fat | 0.071 | 0.290 | 0.617 |
| Tuber | 0.221 | 0.062 | 0.519 |
| Cereals | 0.271 | -0.312 | 0.491 |
| Eggs | -0.284 | 0.149 | 0.412 |
| Alcoholic beverages | -0.048 | 0.044 | 0.371 |
| Cheese | -0.008 | 0.163 | 0.359 |
| Vegetable juice | 0.102 | 0.219 | 0.288 |
| Poultry | -0.364 | -0.324 | 0.268 |
| Seafood | -0.345 | 0.074 | 0.080 |
| Yoghurt | -0.204 | -0.089 | -0.055 |

**T**he dataset’s suitability for PCA was assessed using Bartlett’s test of sphericity, which evaluates the presence of relationships within the data, and the Kaiser-Meyer-Olkin measure, which assesses sampling adequacy. In this study, the Kaiser–Meyer–Olkin measure of sampling adequacy is 0.66, and Bartlett’s test of sphericity is significant (*p ≤* 0*.*0001), indicating the dietary dataset was suitable for principal component analysis.
